# Utility of time in tight range (TITR) in evaluating metabolic control in pediatric and adult patients with type 1 diabetes in treatment with advanced hybrid closed-loop systems

**DOI:** 10.1007/s12020-024-03881-6

**Published:** 2024-05-30

**Authors:** Pilar Bahillo-Curieses, Pablo Fernández Velasco, Paloma Pérez-López, Ana María Vidueira Martínez, María de la O Nieto de la Marca, Gonzalo Díaz-Soto

**Affiliations:** 1 Department of Pediatrics, Pediatric Endocrinology, Hospital Clínico Universitario Valladolid, Ramón y Cajal Avenue, Number 3, 47005 Valladolid, Spain; 2 Department of Endocrinology and Nutrition, Hospital Clínico Universitario Valladolid, Ramón y Cajal Avenue, Number 3, 47005 Valladolid, Spain

**Keywords:** Type 1 diabetes, Time in range, Time in tight range, Advanced hybrid closed loop, Glucose variability

## Abstract

**Purpose:**

To analyze the time in tight range (TITR), and its relationship with other glucometric parameters in patients with type 1 diabetes (T1D) treated with advanced hybrid closed-loop (AHCL) systems.

**Methods:**

A prospective observational study was conducted on pediatric and adult patients with T1D undergoing treatment with AHCL systems for at least 3 months. Clinical variables and glucometric parameters before and after AHCL initiation were collected.

**Results:**

A total of 117 patients were evaluated. Comparison of metabolic control after AHCL initiation showed significant improvements in HbA1c (6.9 ± 0.9 vs. 6.6 ± 0.5%, *p* < 0.001), time in range (TIR) (68.2 ± 11.5 vs. 82.5 ± 6.9%, *p* < 0.001), TITR (43.7 ± 10.8 vs. 57.3 ± 9.7%, *p* < 0.001), glucose management indicator (GMI) (6.9 ± 0.4 vs. 6.6 ± 0.3%, *p* < 0.001), time below range (TBR) 70–54 mg/dl (4.3 ± 4.5 vs. 2.0 ± 1.4%, *p* < 0.001), and time above range (TAR) > 180 mg/dl (36.0 ± 7.6 vs. 15.1 ± 6.4%, *p* < 0.001). Coefficient of variation (CV) also improved (36.3 ± 5.7 vs. 30.6 ± 3.7, *p* < 0.001), while time between 140–180 mg/dl remained unchanged. In total, 76.3% achieved TITR > 50% (100% pediatric). Correlation analysis between TITR and TIR and GRI showed a strong positive correlation, modified by glycemic variability.

**Conclusions:**

AHCL systems achieve significant improvements in metabolic control (TIR > 70% in 93.9% patients). The increase in TIR was not related to an increase in TIR 140–180 mg/dl. Despite being closely related to TIR, TITR allows for a more adequate discrimination of the achieved control level, especially in a population with good initial metabolic control. The correlation between TIR and TITR is directly influenced by the degree of glycemic variability.

## Introduction

In the last century, there have been continuous advances in the treatment of diabetes, with technological advancements gaining special relevance in recent decades. One of the most notable technological milestones is the interstitial glucose monitoring (IGM), which can be considered the most advanced method for monitoring glucose levels in individuals with type 1 diabetes (T1D), recently extending its recommendation to patients with type 2 diabetes (T2D) [1]. The use of IGM is widespread and has brought about a shift in the way diabetes is monitored and understood. Since the introduction of IGM, glucose metric parameters have been established to define the glycemic control of patients with diabetes, previously only defined by HbA1c and capillary blood glucose measurements (SMBG) [2]. The International Consensus on Continuous Glucose Monitoring (CGM) introduced concepts now widely used, such as time in range (TIR), time below range (TBR), time above range (TAR), average glucose, standard deviation (SD), and coefficient of variation (CV) [2]. Subsequently, the International TIR Consensus set control goals for these parameters based on the type of diabetes [3]. In contrast with HbA1c conventional measure, TIR provides a measure of dispersion and allows differentiation between individuals with high and low glucose variability, even when HbA1c levels are similar [4, 5]. The use of TIR has become widespread, but there are doubts about whether it will replace HbA1c, which has been the gold standard for many decades, with studies correlating its levels and the long-term risk of complications [6].

Within this technological advance in diabetes, the integration of CGM with insulin pumps has led to the emergence of advanced hybrid closed-loop systems (AHCL), which have achieved a significant improvement in metabolic control and quality of life for both pediatric and adult patients with T1D [7–13]. Numerous studies have demonstrated their efficacy in treating T1D [7–13].

In recent years, new glucose metric parameters have emerged based on the use of these systems, attempting to address the limitations of previous metrics. One of these is the glucose risk index (GRI), described in 2022 [14]. This new parameter aims to summarize the overall quality of glycemic control for a specific patient in a single figure, simplifying data analysis [14–16]. Another recently introduced and increasingly important metric is the time in tight range (TITR), representing a narrower and more physiological range between 70 and 140 mg/dl. Since the TIR consensus of 2019, there has been discussion regarding the need to adjust glucose ranges, as the glucose ranges and treatment goals were determined through consensus efforts to align with definitions used before the introduction of CGM but were not based on clinical outcomes [3]. Thus, the therapeutic goal of an HbA1c < 7% was equated to spending 70% in the range of 70–180 mg/dl [3]. However, studies showed that individuals without diabetes spend 96% of their time between 70 and 140 mg/dl, rarely reaching levels between 140 and 180 mg/dl, and if they do, it is only for a short period after meals [17, 18]. Based on this, some authors have proposed that TITR may better reflect the CGM metrics of euglycemia, and the first TITR studies have already been published, with some of them indicating desirable TITR goals to define good metabolic control and establishing equivalencies between TITR levels and HbA1c figures [19, 20].

The objective of this study was to analyze the utility and evolution of TITR and its relationship with other glucose metric parameters in patients with T1D (both adults and pediatric) undergoing treatment with AHCL systems, receiving follow-up in a tertiary hospital.

## Material and methods

This study was a prospective observational study involving 117 adult and pediatric patients with T1D undergoing intensive insulin treatment with the Medtronic MiniMed-780G (MM780G) AHCL system at a tertiary hospital. All the patients included in the study participated in an educational program of 3–5 sessions (depending on the previous system used) in which they were instructed about the ACHL system working. Clinical and metabolic control data were collected, and information on system usage and metabolic control was assessed through the data analysis obtained from the Carelink System and Libreview software programs. All patients with T1D and this AHCL system with a scheduled appointment in 2023 were consecutively enrolled, excluding those using the AHCL system less than 3 months or less than 1 year since the onset of DM1. None of the patients met the exclusion criteria. Clinical characteristics and baseline metabolic control status were collected prior to initiating AHCL MM780G treatment. Subsequently, treatment was changed to AHCL MM780G, and metabolic control data were collected during visits conducted in 2023, provided there was at least 3 months of device usage. Metabolic control evaluation involved analyzing glucometric data from device downloads in the 14 days preceding the patient visits.

Glucometric data included mean glucose (mg/dl), glucose management indicator (GMI) (%), TIR (% of time with glucose levels between 70 and 180 mg/dl), TAR (% of time above 180 mg/dl), and TBR (% of time below 70 mg/dl). Glycemic variability was assessed through the CV (%) and SD (mg/dl). TAR and TBR were further classified into very low glycemia level (<54 mg/dL), low glycemia level (54–70 mg/dL), high glycemia level (181–250 mg/dL), and very high glycemia level (>250 mg/dL). Additionally, TITR (% of time between 70 and 140 mg/dl) and time between 140 and 180 mg/dl were calculated, as well as GRI as previously described [15]. Data on the use of AHCL (time in AHCL), total insulin dose, insulin boluses dose and sensor usage percentage were also collected. A comparative analysis was conducted for all these parameters before and after the initiation of the AHCL system.

Statistical analysis was conducted, and the results were expressed as mean ± standard deviation (mean ± SD). The normal distribution of variables was assessed using the Kolmogorov–Smirnov test. Quantitative variables with normal distribution were analyzed using a two-tailed t-Student test, while non-parametric variables were evaluated with the Mann–Whitney U test. Categorical variables were assessed using the chi-square test or Fisher exact test when necessary. Pearson’s linear correlation coefficient was used to analyze the association of quantitative variables. Finally, a multiple linear regression model was employed, with pediatric/adult status, gender, AHCL duration, GMI, CV, time in AHCL, sensor usage, and TIR as independent variable and TITR as the dependent variable. *P* values < 0.05 were considered significant. SPSS 23.0 (SPSS Inc., Chicago, IL, USA) was used for data analysis.

All patients signed an informed consent for their inclusion before participating in the study. The protocol was approved by the Clinical Research Ethics Committee of our Institution (PI 23-3134), and the study was conducted in accordance with the Declaration of Helsinki.

## Results

A total of 117 patients (66% females) were evaluated, including 44 pediatric patients (under 18 years old). Flash glucose monitoring (FGM) was used by 93.2% of the participants, and 82.9% used subcutaneous insulin infusion system (CSII) before transitioning to MM780G AHCL system. Among adults, 9.6% used Medtronic Minimed-670G, and 90.4% used CSII (Medtronic Minimed-640G) with FGM before starting MM780G AHCL system. In the pediatric population, 45.5% received treatment with multiple daily insulin doses (MDI) before transitioning to AHCL, while the rest used Medtronic Minimed-670G (13%) or Medtronic Minimed-640G with FGM (41.5%). The mean age in adults was 44.1 ± 12.6 years, and in pediatric population was 12.77 ± 3.47 years. The mean duration from the start of treatment with the MM780G AHCL system to data analysis was 15.2 ± 9.9 months. In total, 76.1% of the analyzed population had the optimal configuration of the device (MM780G AHCL system): 100 mg/dL target and 2 h of active insulin (see Table 1 for descriptive information).

**Table 1:** Baseline characteristics and evolution after the initiation of treatment with AHCL systems in the entire population

	Basal		MM780G		*p* value
	Mean	SD	Mean	SD	
Gender (female)		66%	–	–	–
Time using MM780G (months)			15.2	9.9	–
HbA1c (%)	6.9	0.9	6.6	0.5	<0.01
Sensor use (%)	91.1	13.5	93.1	5.9	ns
Mean glucose (mg/dl)	150.1	18.8	138.5	13.2	<0.01
GMI (%)	6.9	0.4	6.6	0.3	<0.01
CV (%)	36.3	5.7	30.6	3.7	<0.01
TIR (%)	68.2	11.5	82.5	6.9	<0.01
TBR70 mg/dl (%)	4.3	4.5	2.0	1.4	<0.01
TBR54 mg/dl (%)	1.2	5.9	0.4	0.7	ns
TAR180 mg/dl (%)	36.0	7.6	15.1	6.4	<0.01
TAR250 mg/dl (%)	6.0	5.4	2.1	3.0	<0.01
TITR (%)	43.7	10.8	57.3	9.7	<0.01
TAR140–180 mg/dl (%)	24.6	6.0	24.9	5.6	ns
GRI (%)	37.9	13.0	21.1	10.3	<0.01
Time in AHCL (%)	–	–	92.3	16.0	–
Total insulin dose (UI)	–	–	49.6	65.3	–
Insulin bolus (%)	–	–	39.1	21.3	–
Autocorrection boluses (%)	–	–	26.0	12.4	–

*GMI* glucose management index, *CV* coefficient of variation, *TIR* time in range, *TBR* time below range, *TAR* time above range, *TITR* time in tight range, *GRI* glucose risk index

A comparative study was conducted between glucometric results before and after the initiation of the MM780G AHCL system, with a 3-month minimum usage. Significant improvements were observed in HbA1c (6.9 ± 0.9 vs. 6.6 ± 0.5%, *p* < 0.001), TIR (68.2 ± 11.5 vs. 82.5 ± 6.9%, *p* < 0.001), TITR (43.7 ± 10.8 vs. 57.3 ± 9.7%, *p* < 0.001), GMI (6.9 ± 0.4 vs. 6.6 ± 0.3%, *p* < 0.001), TBR70–54 mg/dl (4.3 ± 4.5 vs. 2.0 ± 1.4%, *p* < 0.001), TAR > 180 mg/dl (36.0 ± 7.6 vs. 15.1 ± 6.4%, *p* < 0.001), and CV (36.3 ± 5.7 vs. 30.6 ± 3.7, *p* < 0.001). The time between 140 and 180 mg/dl remained stable (24.6 ± 6.0 vs. 24.9 ± 5.6, ns) (Table 1). This improvement remained consistent when stratifying by pediatric and adult age groups, with pediatric patients exhibiting better metabolic control for TIR (86.0 ± 4.7 vs. 79.5 ± 7.6%, *p* < 0.001) and TITR (62.7 ± 6.4 vs. 53.7 ± 9.9%, *p* < 0.001) (Table 2).

**Table 2:** Baseline characteristics and evolution after the initiation of treatment with AHCL systems: differences between pediatric population and adults

	Adults		Pediatrics		*p* value
	Mean	SD	Mean	SD	
Time using MM780G (months)	14.5	10.1	16.4	9.6	ns
Basal HbA1c (%)	7.2	0.7	6.5	1.1	<0.01
Basal Sensor use (%)	89.3	5.5	93.5	4.3	ns
Basal mean glucose (mg/dl)	156.5	18.9	141.8	15.1	<0.01
Basal GMI (%)	7.1	0.4	6.7	0.4	<0.01
Basal CV (%)	35.9	5.1	36.9	6.4	ns
Basal TIR (%)	64.5	12.5	72.6	8.5	<0.01
Basal TBR70 mg/dl (%)	3.8	4.9	4.9	4.0	ns
Basal TBR54 mg/dl (%)	1.6	5.4	0.8	0.8	ns
Basal TAR180 mg/dl (%)	23.0	7.9	17.5	6.8	<0.01
Basal TAR250 mg/dl (%)	7.1	5.9	4.6	4.4	<0.01
Basal TITR (%)	40.4	10.3	48.0	10.0	<0.01
Basal TAR140–180 mg/dl (%)	24.7	4.9	24.4	7.2	ns
Basal GRI (%)	40.1	13.7	35.2	11.8	<0.01
Time in AHCL (%)	91.8	12.2	93.3	21.3	ns
Total insulin dose (UI)	54.1	80.0	41.9	22.3	<0.01
Insulin Bolus (%)	25.2	12.6	30.3	3.5	<0.01
Autocorrection boluses (%)	29.4	13.3	20.2	7.9	<0.01
AHCL HbA1c (%)	6.7	0.6	6.4	0.4	<0.01
AHCL Sensor use (%)	91.6	6.7	95.7	2.5	<0.01
AHCL mean glucose (mg/dl)	130.7	8.9	142.9	13.3	<0.01
AHCL GMI (%)	6.7	0.3	6.5	0.2	<0.01
AHCL CV (%)	30.1	3.9	30.3	3.5	ns
AHCL TIR (%)	79.5	7.6	86.0	4.7	<0.01
AHCL TBR70 mg/dl (%)	1.8	1.5	2.5	1.2	<0.01
AHCL TBR54 mg/dl (%)	0.6	2.6	0.5	0.9	ns
AHCL TAR180 mg/dl (%)	15.9	6.6	10.1	3.8	<0.01
AHCL TAR250 mg/dl (%)	2.7	3.5	1.1	1.2	<0.01
AHCL TITR (%)	53.7	9.9	62.7	6.4	<0.01
AHCL TAR140–180 mg/dl (%)	25.8	5.6	23.3	5.4	ns
AHCL GRI (%)	23.2	11.7	17.4	6.0	<0.01

Basal = before starting treatment with MM780 AHCL system. AHCL = in treatment with MM780 AHCL system

*GMI* glucose management index, *CV* coeffient of variation, *TIR* time in range, *TBR* time below range, *TAR* time above range, *TITR* time in tight range, *GRI* glucose risk index, *AHCL* advanced hybrid closed loop, *ns* non significative, *SD* standard deviation

Analyzing the entire sample, 76.3% patients achieved TITR > 50%. However, when considering only the pediatric population, 100% pediatric patients achieved TITR > 50%, compared to 62.5% in the adult population (*p* < 0.001). For TIR > 70%, 93.9% patients reached this goal, with 100% pediatric population and 90.3% adult population achieving it (*p* < 0.001) as well.

**Fig. 1:**
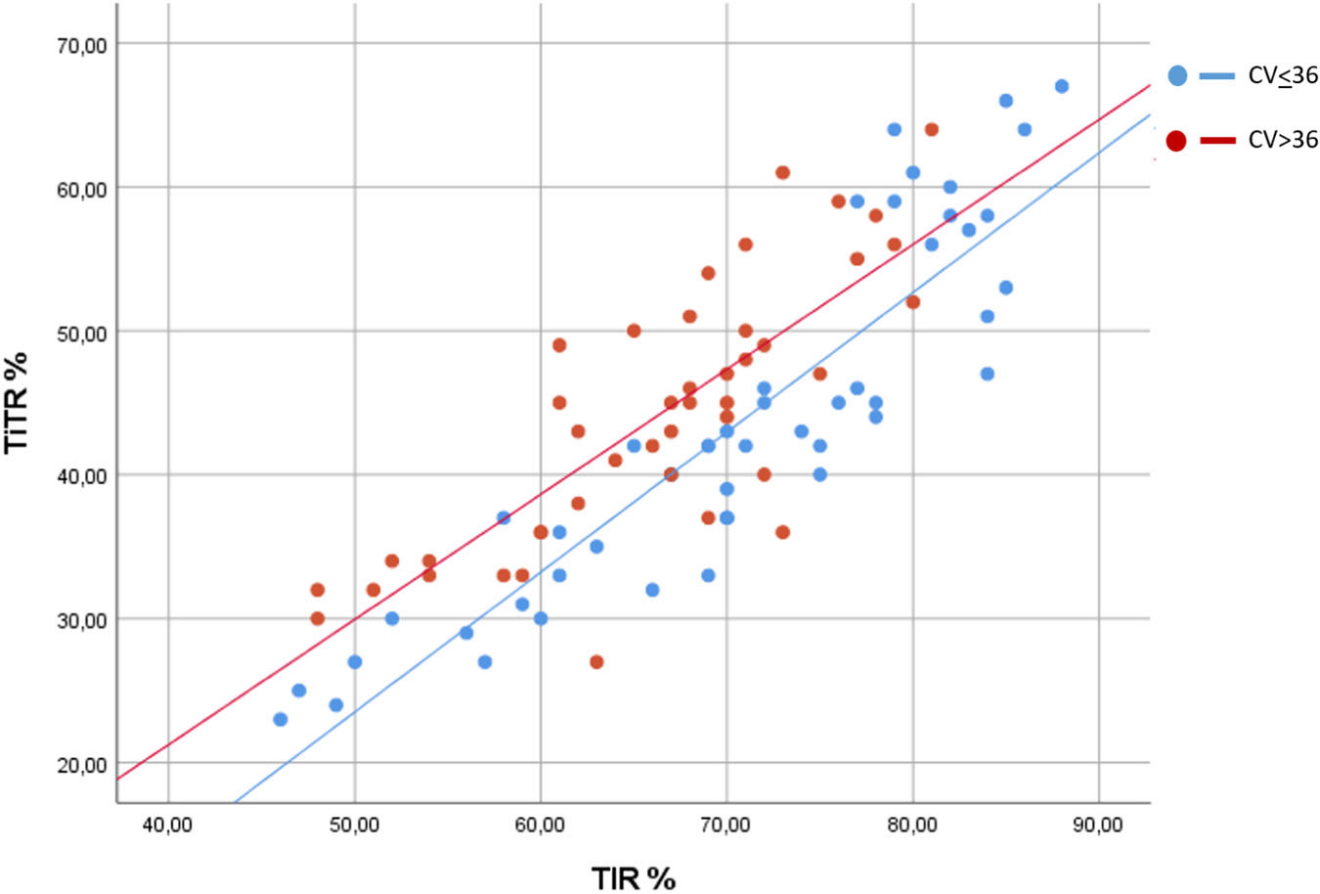
Correlation between time in range (TIR) and time in tight range (TITR) in pediatric and adult patients stratified by coefficient of variation (CV). r = 0.849, *p* < 0.0001

Correlating TITR with TIR revealed a strong positive correlation (r = 0.849, *p* < 0.001) and GRI (r = 0.647, *p* < 0.001), similar in both pediatric and adult populations (Fig. 1). Analyzing the relationship between TIR and TITR and glycemic variability assessed by CV showed that patients with higher variability (CV > 36%) a TIR = 70% corresponded to TITR of 47.9%, compared to those with CV < 36% where a TIR of 70% corresponded to TITR = 42.0%.

In a multivariate analysis evaluating the relationship with TITR among other variables (pediatric/adult status, gender, AHCL duration, GMI, CV, time in AHCL, sensor usage, and TIR), only TIR, CV, and GMI maintained statistical significance (Table 3).

**Table 3:** Multivariate analysis with TITR as the dependent variable

	B	SE	*P* value	CI 95.0%	
Constant	63.891		0.062	−3.263	131.044
Age	0.388	0.020	ns	−1.284	2.060
Gender	−0.731	−0.038	ns	−2.250	0.789
T1D duration	−0.028	−0.031	ns	−0.099	0.043
AHCL GMI	−14.185	−0.406	<0.001	−19.963	−8.406
AHCL CV	0.535	0.216	<0.001	0.246	0.825
AHCL sensor use	0.038	0.016	ns	−0.186	0.262
Time in automatic mode	0.006	0.009	ns	−0.045	0.057
AHCL TIR	0.816	0.628	<0.001	0.556	1.075

*GMI* glucose management index, *CV* coefficient of variation, *TIR* time in range, *AHCL* advanced hybrid closed loop, *B* unstandardized beta coefficient, *SE* standard error, *CI 95.0%* confidence interval 95%

^a^Dependent variable AHCL TITR

## Discussion

The present study demonstrates that treatment with AHCL MM780G system in patients with T1D, both pediatric and adults, improves metabolic control as previously described in prior studies [7–13]. Moreover, this improvement in metabolic control is primarily attributable to an increase in TITR, a recently introduced metric reflecting time spent in euglycemia with limited existing literature, without a change in time spent between 140 and 180 mg/dl. Additionally, we observed a clear correlation between the coefficient of variation (CV) and the relationship between TIR and TITR.

In recent years, the effectiveness of AHCL systems for the treatment of T1D patients, both pediatric and adult, has been demonstrated, leading to a noticeable improvement in their metabolic control [7–13]. It has been established that a TIR > 70% is synonymous with good metabolic control in T1D patients. Studies are now beginning to correlate TIR with macro and microvascular complications, similar to HbA1c levels [21–24]. A new metric, TITR, has been introduced, focusing on a narrower and more physiological range, which is the primary analysis goal in our study. The incorporation of TITR addresses the discussion regarding the need to adjust glucose ranges to values closer to euglycemia, especially considering the significant improvement in glycemic control experienced by individuals with T1D receiving AHCL system treatment. The therapeutic goal of achieving an HbA1c < 7% has been equated to TIR 70%. Some authors propose that TITR may better reflect normal glucose values, with initial studies attempting to establish desirable TITR goals and equivalence between TITR levels and HbA1c figures [19–21].

Our study shows that treatment with AHCL systems results in significant metabolic control improvements for both pediatric and adult patients, assessed not only through TIR but also TITR. The enhancement of metabolic control with AHCL systems, compared to previous treatments, is evident in both HbA1c levels and conventional glucose parameters in our study. Indeed, 93.9% patients achieved a TIR > 70%, considered a parameter for good metabolic control in T1D patients, with 100% pediatric patients achieving this. These results align with the literature, showing an improvement in TIR with a decrease in TBR and TAR, and a betterment in HbA1c levels after initiating treatment with various AHCL systems [7–13].

The main finding of our study is that the improvement in metabolic control with the implementation of AHCL systems primarily occurs due to an increase in TITR, without a corresponding increase in time between 140 and 180 mg/dl, as described by Castañeda et al. [20]. Therefore, treatment with the MM780G AHCL system brings about improvement mainly in time spent in euglycemia, a relevant and significant result not described so far. Our study supports TITR use as a new glucose metric that allows for a more accurate discrimination of the achieved control level, especially in a population with initially good metabolic control and elevated TIR, despite the strong correlation between both parameters.

Although the TITR target is not yet established, it is widely accepted that a high TITR is desirable. A TITR goal > 50% is considered a reasonable and safe treatment target for individuals living with T1D, with higher TITR values being preferable [19, 20]. Peterson et al. established that a 50% TITR corresponds to an HbA1c level of 6.5% [19]. Similarly, Castañeda et al. aim to define TITR levels as treatment goals for individuals with T1D, suggesting that a TITR over 45% has the greatest potential to accurately determine if users achieve an HbA1c below 7% [20]. These authors also state that TITR over 50% is optimal for classifying an GMI below 6.8%, and TITR over 55% is optimal for GMI below 6.5% [20]. Although a TITR over 50% can be considered a reasonable and safe goal for treatment targets in individuals living with T1D, Castañeda et al. showed that a goal exceeding 55% could be reasonably achieved in MM780G AHCL system users if optimal system settings are applied (target 100 mg/dL, insulin duration 2 h). These optimal settings were recorded in 76.1% of our study population.

In our study, metabolic control was better in pediatric population, with an average TITR 62.7 ± 6.4% compared to 53.7 ± 9.9% in adults after starting AHCL. This aligns with Beck et al., who reported an average TITR 53% vs. 32% in non-AHCL users in a mixed population (pediatric and adults) [25]. Castañeda et al. [20] describe that with the use of MM780G AHCL system, TITR significantly increased in both pediatric and adult populations, with an absolute TITR increase of 11.7 ± 10.8% (from 37.2 ± 14.3% to 48.9 ± 9.6%) and 11.6 ± 10.2% (from 37.2 ± 13.6% to 48.8 ± 10.9%), respectively, in each group. Simultaneously, there was a TIR increase of approximately the same magnitude [20]. Therefore, the TITR values described are lower than ours, especially in the younger population [25].

A TITR over 50% was achieved by 76.3% of our patients. When specifically analyzing the pediatric population, 100% pediatric patients achieved a TITR over 50%, in contrast with adult population where only a lower percentage (62.5%) reached this threshold. Nevertheless, Castañeda et al. describe that over 90% users reached a TITR over 45%, considering it a high percentage, similar to what our work considers [20]. Comparing our results with Panassini’s et al. [26], in a study conducted exclusively in pediatric population, the average TITR described is clearly lower than ours (36.4 ± 12.8%). Although this average corresponds to patients with different treatments, they describe a higher TITR in users of AHCL systems where the TITR was 45 ± 11.2%, a percentage clearly lower than that obtained in our pediatric population [26]. On the other hand, the percentage of participants achieving TITR over 50% in the AHCL group was significantly lower than in ours (34.3%).

TITR shows a close correlation with TIR and CV, as shown recently [25] The correlation between TITR and GRI was recently described [15]. The multivariate analysis conducted in our study shows that only TIR, CV and GMI maintained statistical significance with TITR. The influence of glucose variability, not directly included in TITR or TIR, has been described for both variables, partly due to the dependence of CV calculation on mean glucose. However, our study suggests that the TITR target of 50% could be adjusted based on individual glucose variability. In other words, higher CV leads to a higher TITR for a given TIR, just as greater variability for a specific TITR results in lower TIR. This was previously described by Beck et al., who reported that higher CV, and in turn, higher TBR lead to a higher TITR for a given TIR [25]. TBR should also be considered in this relationship between TIR and TITR, as noted by Becks et al., who found differences in the TIR and TITR relationship in patients with T1D and T2D, with a higher TITR for a given TIR in T1D patients [25]. These differences are attributable to higher variability and TBR in T1D patients, and in fact, the differences in the TIR and TITR relationship between both populations disappeared when adjusted for CV or TBR [25].

One of the limitations of our study is the smaller sample size compared to other studies carried out with the same AHCL system. However, it is important to highlight as strengths that, unlike studies with a larger we have real clinical data and an accurate diagnosis of the included patients, sample size, as well as HbA1c data, allowing for an exhaustive comparison of the before and after conditions upon initiating treatment with AHCL systems.

In conclusion, our study highlights that AHCL systems achieve significant improvements in metabolic control with TIR > 70% in 100% pediatric patients, primarily through increases in TITR and, consequently, time spent in euglycemia. Therefore, TITR may be a more suitable target for patients undergoing AHCL system treatment, indicating improved metabolic control. The emerging role of TITR as a key metric for evaluating glucose control in individuals with diabetes may be closely linked to a careful interpretation of CV levels in both research studies and daily clinical practice. Further studies are needed to evaluate short- and long-term outcomes of using TITR as a metric.

## References

[1] M. Yaron, E. Roitman, G. Aharon-Hananel, Z. Landau, T. Ganz, I. Yanuv, A. Rozenberg, M. Karp, M. Ish-Shalom, J. Singer, J. Wainstein, I. Raz, Effect of flash glucose monitoring technology on glycemic control and treatment satisfaction in patients with type 2 diabetes. Diabetes Care **42**(7), 1178–1184 (2019). 10.2337/dc18-0166 31036546 10.2337/dc18-0166

[2] T. Danne, R. Nimri, T. Battelino, R.M. Bergenstal, K.L. Close, J.H. DeVries, S. Garg, L. Heinemann, I. Hirsch, S.A. Amiel, R. Beck, E. Bosi, B. Buckingham, C. Cobelli, E. Dassau, F.J. Doyle 3rd, S. Heller, R. Hovorka, W. Jia, T. Jones, O. Kordonouri, B. Kovatchev, A. Kowalski, L. Laffel, D. Maahs, H.R. Murphy, K. Nørgaard, C.G. Parkin, E. Renard, B. Saboo, M. Scharf, W.V. Tamborlane, S.A. Weinzimer, M. Phillip, International Consensus on use of continuous glucose monitoring. Diabetes Care **40**(12), 1631–1640 (2017). 10.2337/dc17-1600 29162583 10.2337/dc17-1600 PMC6467165

[3] T. Battelino, T. Danne, R.M. Bergenstal, S.A. Amiel, R. Beck, T. Biester, E. Bosi, B.A. Buckingham, W.T. Cefalu, K.L. Close, C. Cobelli, E. Dassau, J.H. DeVries, K.C. Donaghue, K. Dovc, F.J. Doyle 3rd, S. Garg, G. Grunberger, S. Heller, L. Heinemann, I.B. Hirsch, R. Hovorka, W. Jia, O. Kordonouri, B. Kovatchev, A. Kowalski, L. Laffel, B. Levine, A. Mayorov, C. Mathieu, H.R. Murphy, R. Nimri, K. Nørgaard, C.G. Parkin, E. Renard, D. Rodbard, B. Saboo, D. Schatz, K. Stoner, T. Urakami, S.A. Weinzimer, M. Phillip, Clinical targets for continuous glucose monitoring data interpretation: recommendations from the International Consensus on time in range. Diabetes Care **42**(8), 1593–1603 (2019). 10.2337/dci19-0028 31177185 10.2337/dci19-0028 PMC6973648

[4] Z. Bloomgarden, Beyond HbA1c. J. Diabetes **9**(12), 1052–1053 (2017). 10.1111/1753-0407.12590 28792665 10.1111/1753-0407.12590

[5] R.W. Beck, C.G. Connor, D.M. Mullen, D.M. Wesley, R.M. Bergenstal, The fallacy of average: how using HbA_1c_ alone to assess glycemic control can be misleading. Diabetes Care **40**, 994–999 (2017). 10.2337/dc17-0636 28733374 10.2337/dc17-0636 PMC5521971

[6] Diabetes Control and Complications Trial (DCCT)/Epidemiology of Diabetes Interventions and Complications (EDIC) Study Research Group, Intensive diabetes treatment and cardiovascular outcomes in type 1 diabetes: the DCCT/EDIC study 30-yearfollow-up. Diabetes Care **39**(5), 686–693 (2016). 10.2337/dc15-1990 10.2337/dc15-1990 PMC4839174 26861924

[7] J.D. Silva, G. Lepore, T. Battelino, A. Arrieta, J. Castañeda, B. Grossman, J. Shin, O. Cohen, Real-world performance of the MiniMed™ 780G system: first report of outcomes from 4120 users. Diabetes Technol. Ther. **24**(2), 113–119 (2022). 10.1089/dia.2021.0203 34524003 10.1089/dia.2021.0203 PMC8817690

[8] A. Arrieta, T. Battelino, A.E. Scaramuzza, J. Da Silva, J. Castañeda, T.L. Cordero, J. Shin, O. Cohen, Comparison of MiniMed 780G system performance in users aged younger and older than 15 years: evidence from 12 870 real-world users. Diabetes Obes. Metab. **24**(7), 1370–1379 (2022). 10.1111/dom.14714 35403792 10.1111/dom.14714 PMC9545031

[9] J. Kesavadev, A. Basanth, G. Krishnan, A. Shankar, G. Sanal, S. Jothydev, Real-world user and clinician perspective and experience with MiniMed™ 780G advanced hybrid closed loop system. Diabetes Ther. **14**(8), 1319–1330 (2023). 10.1007/s13300-023-01427-z 37278948 10.1007/s13300-023-01427-z PMC10299959

[10] J. Ware, C.K. Boughton, J.M. Allen, M.E. Wilinska, M. Tauschmann, L. Denvir, A. Thankamony, F.M. Campbell, R.P. Wadwa, B.A. Buckingham, N. Davis, L.A. DiMeglio, N. Mauras, R.E.J. Besser, A. Ghatak, S.A. Weinzimer, K.K. Hood, D.S. Fox, L. Kanapka, C. Kollman, J. Sibayan, R.W. Beck, R. Hovorka; DAN05 Consortium, Cambridge hybrid closed-loop algorithm in children and adolescents with type 1 diabetes: a multicentre 6-month randomised controlled trial. Lancet Digit. Health. **4**(4), e245–e255 (2022). 10.1016/S2589-7500(22)00020-6 35272971 10.1016/S2589-7500(22)00020-6

[11] R. Graham, L. Mueller, M. Manning, S. Habif, L.H. Messer, J.E. Pinsker, E. Aronoff-Spencer, Real-world use of control-IQ technology is associated with a lower rate of severe hypoglycemia and diabetic ketoacidosis than historical data: results of the Control-IQ Observational (CLIO) Prospective study. Diabetes Technol. Ther. **26**(1), 24–32 (2024). 10.1089/dia.2023.0341 37782904 10.1089/dia.2023.0341 PMC10794820

[12] R.W. Beck, L.G. Kanapka, M.D. Breton, S.A. Brown, R.P. Wadwa, B.A. Buckingham, C. Kollman, B. Kovatchev, A meta-analysis of randomized trial outcomes for the t:slim X2 insulin pump with control-IQ technology in youth and adults from age 2 to 72. Diabetes Technol. Ther. **25**(5), 329–342 (2023). 10.1089/dia.2022.0558 37067353 10.1089/dia.2022.0558 PMC10171957

[13] M.D. Breton, B.P. Kovatchev, One year real-world use of the control-IQ advanced hybrid closed-loop technology. Diabetes Technol. Ther. **23**(9), 601–608 (2021). 10.1089/dia.2021.0097 33784196 10.1089/dia.2021.0097 PMC8501470

[14] D.C. Klonoff, J. Wang, D. Rodbard, M.A. Kohn, C. Li, D. Liepmann, D. Kerr, D. Ahn, A.L. Peters, G.E. Umpierrez, J.J. Seley, N.Y. Xu, K.T. Nguyen, G. Simonson, M.S.D. Agus, M.E. Al-Sofiani, G. Armaiz-Pena, T.S. Bailey, A. Basu, T. Battelino, S.Y. Bekele, P.Y. Benhamou, B.W. Bequette, T. Blevins, M.D. Breton, J.R. Castle, J.G. Chase, K.Y. Chen, P. Choudhary, M.A. Clements, K.L. Close, C.B. Cook, T. Danne, F.J. Doyle 3rd, A. Drincic, K.M. Dungan, S.V. Edelman, N. Ejskjaer, J.C. Espinoza, G.A. Fleming, G.P. Forlenza, G. Freckmann, R.J. Galindo, A.M. Gomez, H.A. Gutow, L. Heinemann, I.B. Hirsch, T.D. Hoang, R. Hovorka, J.H. Jendle, L. Ji, S.R. Joshi, M. Joubert, S.K. Koliwad, R.A. Lal, M.C. Lansang, W.A. Lee, L. Leelarathna, L.A. Leiter, M. Lind, M.L. Litchman, J.K. Mader, K.M. Mahoney, B. Mankovsky, U. Masharani, N.N. Mathioudakis, A. Mayorov, J. Messler, J.D. Miller, V. Mohan, J.H. Nichols, K. Nørgaard, D.N. O’Neal, F.J. Pasquel, A. Philis-Tsimikas, T. Pieber, M. Phillip, W.H. Polonsky, R. Pop-Busui, G. Rayman, E.J. Rhee, S.J. Russell, V.N. Shah, J.L. Sherr, K. Sode, E.K. Spanakis, D.J. Wake, K. Waki, A. Wallia, M.E. Weinberg, H. Wolpert, E.E. Wright, M. Zilbermint, B. Kovatchev, A glycemia risk index (GRI) of hypoglycemia and hyperglycemia for continuous glucose monitoring validated by clinician ratings. J. Diabetes Sci. Technol. **17**(5), 1226–1242 (2023). 10.1177/19322968221085273 35348391 10.1177/19322968221085273 PMC10563532

[15] G. Díaz-Soto, P. Pérez-López, P. Férnandez-Velasco, M.O. Nieto de la Marca, E. Delgado, S. Del Amo, D. de Luis, P. Bahillo-Curieses, Glycemia risk index assessment in a pediatric and adult patient cohort with type 1 diabetes mellitus. J. Diabetes Sci. Technol. (2023). 10.1177/19322968231154561 10.1177/19322968231154561 PMC11418463 36794818

[16] P. Pérez-López, P. Férnandez-Velasco, P. Bahillo-Curieses, D. de Luis, G. Díaz-Soto, Impact of glucose variability on the assessment of the glycemia risk index (GRI) and classic glycemic metrics. Endocrine **82**(3), 560–568 (2023). 10.1007/s12020-023-03511-7 37695452 10.1007/s12020-023-03511-7 PMC10618378

[17] V.N. Shah, S.N. DuBose, Z. Li, R.W. Beck, A.L. Peters, R.S. Weinstock, D. Kruger, M. Tansey, D. Sparling, S. Woerner, F. Vendrame, R. Bergenstal, W.V. Tamborlane, S.E. Watson, J. Sherr, Continuous glucose monitoring profiles in healthy nondiabetic participants: a multicenter prospective study. J. Clin. Endocrinol. Metab. **104**(10), 4356–4364 (2019). 10.1210/jc.2018-02763 31127824 10.1210/jc.2018-02763 PMC7296129

[18] O. Cohen, R. Basu, G. Bock, C. Dalla Man, M. Campioni, A. Basu, G. Toffolo, C. Cobelli, R.A. Rizza, Prediction of postprandial glycemic exposure: utility of fasting and 2-h glucose measurements alone and in combination with assessment of body composition, fitness, and strength. Diabetes Care **29**(12), 2708–2713 (2006). 10.2337/dc06-1118 17130209 10.2337/dc06-1118

[19] J. Petersson, K. Åkesson, F. Sundberg, S. Särnblad, Translating glycated hemoglobin A1c into time spent in glucose target range: a multicenter study. Pediatr. Diabetes **20**(3), 339–344 (2019). 10.1111/pedi.12817 30652407 10.1111/pedi.12817

[20] J. Castañeda, A. Arrieta, T. van den Heuvel, T. Battelino, O. Cohen, Time in tight glucose range in type 1 diabetes: predictive factors and achievable targets in real-world users of the MiniMed 780G system. Diabetes Care. (2023). 10.2337/dc23-1581 10.2337/dc23-1581 PMC11043222 38113453

[21] R.W. Beck, R.M. Bergenstal, T.D. Riddlesworth, C. Kollman, Z. Li, A.S. Brown, K.L. Close, Validation of time in range as an outcome measure for diabetes clinical trials. Diabetes Care **42**(3), 400–405 (2019). 10.2337/dc18-1444 30352896 10.2337/dc18-1444 PMC6905478

[22] R.M. Bergenstal, E. Hachmann-Nielsen, K. Kvist, A.L. Peters, J.M. Tarp, J.B. Buse, Increased derived time in range is associated with reduced risk of major adverse cardiovascular events, severe hypoglycemia, and microvascular events in type 2 diabetes: a post hoc analysis of DEVOTE. Diabetes Technol. Ther. **25**(6), 378–383 (2023). 10.1089/dia.2022.0447 37017470 10.1089/dia.2022.0447 PMC10398723

[23] R.W. Beck, The association of time in range and diabetic complications: the evidence is strong. Diabetes Technol. Ther. **25**(6), 375–377 (2023). 10.1089/dia.2023.0141 36971584 10.1089/dia.2023.0141

[24] V.N. Shah, L.G. Kanapka, H.K. Akturk, S. Polsky, G.P. Forlenza, C. Kollman, R.W. Beck, J. Snell-Bergeon, Time in range is associated with incident diabetic retinopathy in adults with type 1 diabetes: a longitudinal study. Diabetes Technol. Ther. (2023). 10.1089/dia.2023.0486 10.1089/dia.2023.0486 38133643

[25] R.W. Beck, D. Raghinaru, P. Calhoun, R.M. Bergenstal, A comparison of continuous glucose monitoring-measured time-in-range 70-180 mg/dL versus time-in-tight-range 70-140 mg/dL. Diabetes Technol. Ther. (2023). 10.1089/dia.2023.0380 10.1089/dia.2023.0380 37870460

[26] S. Passanisi, C. Piona, G. Salzano, M. Marigliano, B. Bombaci, A. Morandi, A. Alibrandi, C. Maffeis, F. Lombardo, Aiming for the best glycemic control beyond time in range: time in tight range as a new CGM metric in children and adolescents with type 1 diabetes using different treatment modalities. Diabetes Technol. Ther. (2023). 10.1089/dia.2023.0373 10.1089/dia.2023.0373 37902743

